# Visual evoked potential abnormalities in spinocerebellar ataxia type 27B: a case report

**DOI:** 10.1007/s10633-026-10111-z

**Published:** 2026-05-11

**Authors:** Pavol Skacik, Stefan Sivak, Milan Grofik, Egon Kurca

**Affiliations:** Neurology Department Jessenius Faculty of Medicine and University Hospital Martin, Kollarova 2, 036 01 Martin, Slovakia

**Keywords:** Spinocerebellar ataxia type 27B, FGF14 repeat expansion, Visual evoked potentials, Cerebellar ataxia, Visual pathway involvement

## Abstract

**Introduction:**

Spinocerebellar ataxia type 27B (SCA27B) is an autosomal dominant late-onset cerebellar ataxia caused by a pathogenic GAA repeat expansion in the *FGF14* gene. Although oculomotor abnormalities, particularly downbeat nystagmus, are well recognized, afferent visual pathway involvement has not been systematically investigated.

**Case presentation:**

We report a 67-year-old man with genetically confirmed SCA27B who presented with a two-year history of progressive cerebellar and somatosensory ataxia, with a Scale for the Assessment and Rating of Ataxia (SARA) score of 13. Neurological examination revealed typical oculomotor abnormalities, including downbeat nystagmus elicited during head-shaking testing. Brain MRI showed mild cerebellar atrophy. Electrophysiological studies demonstrated axonal sensory polyneuropathy and prolonged central conduction times on somatosensory evoked potentials. Comprehensive ophthalmological examination, including optical coherence tomography, showed no structural abnormalities. Pattern-reversal visual evoked potentials, recorded according to ISCEV standards, demonstrated mild bilateral prolongation of P100 latency, measuring 116 ms in the left eye and 115 ms in the right eye, with preserved amplitudes. Compared with laboratory normative data from individuals aged 60–70 years, these values exceeded the upper age-related reference limit.

**Conclusion:**

This case suggests possible subclinical functional involvement of the post-retinal afferent visual pathways in SCA27B. However, the findings should be interpreted cautiously and confirmed in larger, age-matched cohorts.

## Introduction

Spinocerebellar ataxia type 27B (SCA27B) is increasingly recognized as one of the most common genetic causes of late-onset cerebellar ataxia. It is inherited in an autosomal dominant manner and is associated with a pathogenic expansion of ≥ 250 GAA repeats within intron 1 of the *FGF14* gene [1]. Clinically, SCA27B typically presents with a slowly progressive cerebellar syndrome beginning in adulthood, commonly manifesting as cerebellar ataxia, dysarthria, and characteristic oculomotor abnormalities, particularly downbeat nystagmus. In addition, many patients may experience episodic neurological symptoms alongside the chronic ataxic syndrome [2].

Electrophysiological studies have demonstrated variable patterns of involvement across different spinocerebellar ataxia (SCA) subtypes. In particular, visual evoked potentials (VEPs) in patients with SCA1, SCA2, SCA3, SCA6, SCA7 have shown abnormalities such as prolonged P100 latencies or reduced amplitudes [3].

These observations suggest that electrophysiological testing may provide insights into pathway involvement and may help delineate phenotypic characteristics across different SCA subtypes, as well as contribute to the assessment of disease extent and progression. Nevertheless, considerable overlap in electrophysiological findings between different SCAs is frequently observed [3]. Importantly, electrophysiological evaluation of the visual system may detect functional abnormalities along the afferent visual pathways even in the absence of structural changes on routine ophthalmological examinations, such as slit-lamp evaluation or optical coherence tomography (OCT).

In the present case report, we describe abnormal VEP findings in a patient with genetically confirmed SCA27B, which may indicate possible subclinical visual pathway involvement and warrants further investigation in larger cohorts.

### Case presentation

A 67-year-old male with genetically confirmed SCA27B presented with a progressive clinical picture characterized by combined cerebellar and somatosensory ataxia. The age at symptom onset was 65 years, and the disease duration at the time of evaluation was 2 years. The severity of ataxia, assessed using the Scale for the Assessment and Rating of Ataxia (SARA), was 13. The patient did not report episodic neurological symptoms. Genetic testing confirmed the presence of a pathogenic GAA repeat expansion in the *FGF14* gene.

Extensive diagnostic evaluation, including laboratory testing, cerebrospinal fluid analysis, and neuroimaging, excluded alternative or concomitant etiologies. Brain MRI demonstrated mild global cerebellar atrophy without other significant structural abnormalities.

Video-oculographic examination revealed downbeat nystagmus elicited during the head-shaking test, frequent square-wave jerks, and impaired saccadic and smooth pursuit eye movements. Vestibular testing showed mild bilateral vestibular hypofunction, predominantly affecting the vertical semicircular canals. Electromyography confirmed the presence of axonal sensory polyneuropathy. Somatosensory evoked potentials (SSEPs) of the median and tibial nerves demonstrated prolonged central conduction times, suggesting involvement of the central somatosensory pathways.

Comprehensive ophthalmological assessment demonstrated normal visual acuity. Slit-lamp examination showed no evidence of cataract, and fundoscopic examination revealed no abnormalities of the retina or optic disc. OCT measurements were within normal limits in both eyes, with no structural retinal abnormalities detected.

Pattern-reversal VEP were performed to assess possible functional involvement of the afferent visual pathways. Recordings were obtained monocularly, with the fellow eye occluded, using full-field black-and-white checkerboard stimulation presented on an LCD monitor at a viewing distance of 100 cm in a dimly lit room. The stimulus field subtended approximately 15° of visual angle. Central fixation was maintained, and habitual optical correction was applied when required. The checkerboard stimulus had a check size of 1°, a mean luminance of 50 cd/m^2^, and a Michelson contrast of 80%. Pattern reversal was performed at a rate of 2 reversals/s, with an acquisition time of 300 ms. Electrodes were positioned according to the international 10–10 system, with the active electrode at Oz, the reference electrode at Fz, and the ground electrode on the forehead. Signals were recorded from the Oz–Fz derivation using a band-pass filter of 1–50 Hz. Reproducibility was confirmed by repeated recordings for each eye. VEP recordings were conformed to the ISCEV standards [4].

**Fig. 1:**
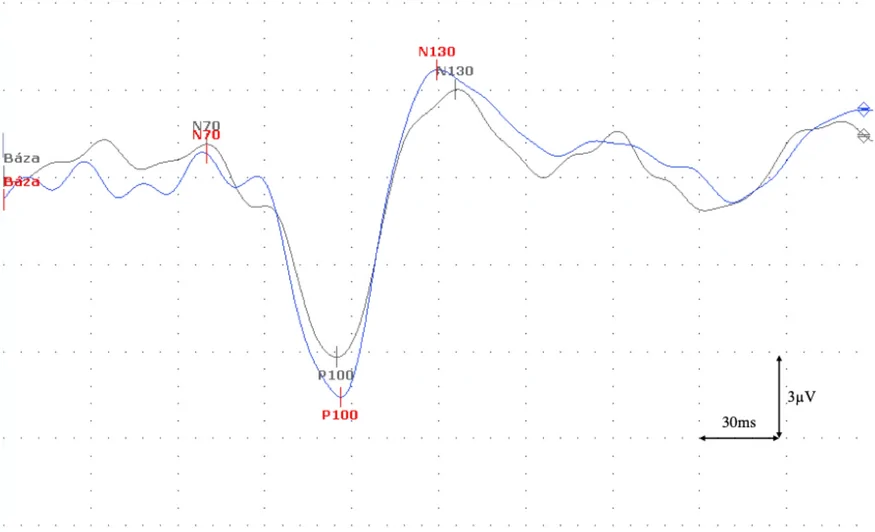
Pattern-reversal full-field VEPs in a patient with genetically confirmed SCA27B. Bilaterally mildly prolonged P100 latencies are present with preserved amplitudes. P100 latency was 116 ms in the left eye and 115 ms in the right eye, exceeding age-related laboratory normative values for individuals 60 to 70 years of age. VEP amplitudes were 8.4 µV in the left eye and 7.4 µV in the right eye, both within the normal range. Blue trace: left-eye VEP; gray trace: right-eye VEP

The principal abnormality observed on VEP testing was mild bilateral prolongation of P100 latency. P100 latency measured 116 ms in the left eye and 115 ms in the right eye, while VEP amplitudes were preserved (Fig. 1). For comparison, laboratory reference data from individuals aged 60–70 years showed a mean P100 latency of 106.8 ms, with an estimated 2.5th–97.5th percentile reference range of 99.0–111.1 ms for pooled left- and right-eye recordings. The patient’s P100 latencies therefore exceeded the upper age-related reference limit bilaterally, supporting the interpretation of mild bilateral P100 prolongation.

These findings may indicate possible functional involvement of the post-retinal afferent visual pathways. However, they should be interpreted cautiously given the single-case design and the potential influence of age-related latency prolongation and other confounding factors.

## Discussion

Electrophysiological testing as part of biomarkers has been evaluated in patients with various SCAs at both preclinical and clinical stages; however, longitudinal data remain limited in many disorders [5]. Most published studies have focused primarily on electromyography and nerve conduction studies, often in relation to polyneuropathy or sensory tract involvement, as well as on motor evoked potentials [6]. In contrast, involvement of the visual system has been investigated in other hereditary ataxias, particularly Friedreich ataxia, where structural and functional abnormalities of the visual pathways have been described and shown to correlate with disease severity and progression of neurological disability [7].

Regarding neuro-ophthalmological involvement, previous studies in SCA27B have mainly focused on video-oculographic characteristics describing typical oculomotor abnormalities, particularly downbeat nystagmus and related findings [8, 9]. In our case, VEP findings suggested possible subclinical involvement of the afferent visual pathways despite the absence of structural ophthalmological abnormalities.

In several other spinocerebellar ataxia subtypes, involvement of the visual system has been reported, including structural and functional abnormalities affecting the retina and optic nerves [3]. These observations suggest that the neurodegenerative process in hereditary ataxias may extend beyond the cerebellar system to involve additional neural pathways. In this context, the prolonged P100 latencies observed in our patient may reflect subclinical involvement of the visual pathways, even in the absence of visual symptoms or detectable structural abnormalities on ophthalmological examination.

Eye movement abnormalities may potentially influence the reliability of pattern-reversal VEP recordings. Fixation instability and retinal image motion associated with nystagmus may affect response morphology, particularly by reducing P100 amplitudes and increasing response variability. These effects have been described mainly in congenital or early-onset forms of nystagmus, particularly horizontal types, whereas evidence specifically addressing downbeat nystagmus remains limited [10, 11]. Importantly, latency parameters are generally considered less susceptible to the effects of eye movements than amplitude measures [12]. In our patient, no spontaneous nystagmus was observed during VEP testing, and downbeat nystagmus was elicited only during the head-shaking test during oculomotor examination. Therefore, although a potential influence of eye movement instability cannot be completely excluded, it is unlikely to fully explain the observed bilateral prolongation of P100 latency.

Ophthalmological factors such as reduced visual acuity, cataract, or retinal pathology may also influence VEP waveforms [12]. However, comprehensive ophthalmological examination in our patient revealed no structural abnormalities. It should also be noted that physiological aging may be associated with mild prolongation of VEP P100 latency, representing a potential confounding factor when interpreting electrophysiological findings in elderly patients [13]. Age-related latency prolongation is relatively common and may overlap with disease-related abnormalities, particularly in older patients with ataxias, in whom delayed VEPs may partly reflect physiological aging rather than specific involvement of the visual pathways. Therefore, VEP results should be interpreted using age-adjusted normative data derived from appropriately recruited age-specific control groups. To ensure reliable interpretation and reproducibility, each laboratory should establish its own reference ranges for relevant age groups under its specific recording conditions [4].

In conclusion, this observation suggests that electrophysiological assessment may provide added value in the evaluation of hereditary ataxias by detecting functional pathway abnormalities that may not be apparent on structural neuroimaging or routine ophthalmological examination. In SCA27B, where visual pathway involvement has not yet been systematically investigated, abnormal VEP findings may indicate broader neurophysiological involvement beyond the clinically dominant cerebellar syndrome. More broadly, VEPs and other electrophysiological modalities may help refine our understanding of the complexity and extent of pathway involvement in hereditary ataxias, complementing clinical and imaging-based assessments. To our knowledge, this is the first report describing VEP abnormalities in a genetically confirmed patient with SCA27B.

### Limitations and future directions

Given the descriptive nature of this single-case report, the findings should be interpreted with caution. Several factors may influence VEP recordings, including age-related changes in latency, visual acuity, refractive error, ocular media opacity, retinal or optic nerve pathology, fixation instability, reduced attention or cooperation during testing, technical variability, and differences in stimulation or recording parameters. Although no major ophthalmological abnormality was identified, subtle or subclinical visual pathway changes cannot be fully excluded. Larger studies including genetically confirmed SCA27B patients and appropriately age-matched controls are needed to determine the prevalence, specificity, and clinical relevance of visual pathway involvement, and to clarify the potential role of VEPs as a neurophysiological biomarker in this disorder.

## Data Availability

The datasets generated and/or analyzed during the current study are available from the corresponding author on reasonable request.
